# Efficacy of *Camellia sinensis* extract against *Candida* species in patients with denture stomatitis

**DOI:** 10.18502/cmm.4.3.174

**Published:** 2018-09

**Authors:** Anahita Ghorbani, Ashena Sadrzadeh, Emran Habibi, Kosar Dadgar, Jafar Akbari, Mahmood Moosazadeh, Bakhshi Hossein, Fatemeh Ahangarkani, Afsane Vaezi

**Affiliations:** 1 Department of Oral and Maxillofacial Medicine, Faculty of Dentistry, Mazandaran University of Medical Sciences, Sari, Iran; 2 School of Dentistry, Mazandaran University of Medical Sciences, Sari, Iran; 3 Department of Pharmacognosy, Pharmaceutical Sciences Research Center, Faculty of Pharmacy, Mazandaran University of Medical Sciences, Sari, Iran; 4 Department of Prosthodontics, Faculty of Dentistry, Mazandaran University of Medical Sciences, Sari, Iran; 5 Department of Pharmaceutics, Faculty of Pharmacy, Mazandaran University of Medical Sciences, Sari, Iran; 6 Health Sciences Research Center, Addiction Institute, Mazandaran University of Medical Sciences, Sari, Iran; 7 Department of Pharmacognosy, School of Pharmacy, Mazandaran University of Medical Sciences, Sari, Iran; 8 Department of Medical Mycology, Mazandaran University of Medical Sciences, Sari, Iran; 9 Student Research Committee, Mazandaran University of Medical Sciences, Sari, Iran

**Keywords:** Camellia sinensis, Candidiasis, Denture stomatitis, Green tea, Nystatin

## Abstract

**Background and Purpose:**

Denture stomatitis is a chronic inflammation disease of the oral mucosa, which is specified by erythematous lesions mainly in the upper palate. Nystatin as a polyene, a class of antifungal agents, is one of the effective drugs to treat denture stomatitis. Considering the expansion of utilizing herbal drugs to cure many kinds of diseases, the present study was conducted to investigate the effects of *Camellia sinensis* (green tea), which has the most chemical and influence similarity with nystatin, against denture stomatitis.

**Materials and Methods:**

This study was conducted on 22 patients with a positive mycological evidence for denture stomatitis caused by *Candida* species. The study population was divided into two groups, namely green tea and nystatin, receiving green tea mouthwash 0.5% and nystatin suspension 100,000 U/ml, respectively. The lesion size and number of yeast colonies were measured before and after the treatment.

**Results:**

According to the results, both groups showed reduced lesion size, clinical improvement, and significant reduction of *Candida* colony count in both group of patients were showedafter the therapeutic. Based on the results of polymerase chain reaction, *Candida albicans* was the most common species isolated from denture stomatitis. There was no significant difference between the two study groups in terms of *Candida* species distribution (*P=0.700*).

**Conclusion:**

Green tea demonstrated a comparable anti-*Candida* activity with regard to nystatin; therefore, it could be recommended as an alternative treatment.

## Introduction

Denture stomatitis is a chronic inflammatory disease of the oral mucosa, which is characterized by erythematous lesions mainly in the upper palate. This inflammation may induce chronic erythema, palatal inflammation, pain, and sense of burning [1, 2]. The most common causes of denture stomatitis are opportunist fungal infections, such as oral chronic candidiasis (11-67%) [1, 2]. This infection has a multifactorial etiology, and its main risk factors include denture trauma, use of denture, poor sanitation, systemic diseases, cancer, use of antibiotics and corticosteroids, diabetes, and exposure to immunosuppression therapy. 

*Candida albicans* is known as the most common organism in denture stomatitis pathogenesis [3]. Different antifungal drugs such as poleyene (nystatin), imidazole (clotrimazole), and chlorhexidine, are used to treat denture stomatitis. Although these drugs are effective against this infection, undesirable side effects should be considered. In this regard, oral nystatin leaves an unpleasant taste and its topical form may lead to allergic reactions. Moreover, the aforementioned medications are reported to result in adrenal failure, hepatic necrosis, and drug resistance [4]. 

Green tea (*Camellia sinensis *(L.) Kuntze) shows antifungal activity against *Candida* species due to its polyphenolic ingredients [5]. The difference between black and green tea is in the operation and fermentation process. They are also different in phenolic compounds and caffeine degree [6]. Tannin polyphenol as an important metabolite of tea, with high molecular mass, has the capability to combine with other big molecules, such as proteins, alkaloids, cellulose, starch, and heavy metals [7]. 

In the current decades, the potential antimicrobial, anticarcinogenic, antimutagenic, and especially antidiarrheal properties of this polyphenols are significantly considered [8]. Regarding this, the aim of this study was to assess the efficacy and anti-*Candida* activity of green tea mouthwash (0.5%), compared to those of nystatin suspension 100,000 U/ml, in the treatment of denture stomatitis through the quantification of isolated *Candida* species of mucosa before and after the treatment.

## Materials and Methods

***Patients***

This study was conducted on 22 patients with denture stomatitis referring to the Department of Oral and Dental Diseases affiliated with Mazandaran University of Medical Sciences, Sari, Iran. The exclusion criteria were: 1) use of antifungal, antibiotic, and anti-steroid agents in the recent months, 2) allergic reaction to nystatin, 3) immune dysfunction or Alzheimer’s disease, 4) psychological problems, 5) problems in the muscles of mastication, and 6) improper denture or color change in denture. 

***Plant material and herbal mouthwash preparation***

The green tea mouthwash 0.5% [9] was produced by the Department of Pharmacognosy, Faculty of Pharmacy, Mazandaran University of Medical Sciences. The leaves of tea (*Camellia sinensis L*) were obtained from Guilan, Iran. The collected material was shade dried for 6 days, and then converted to coarse powder. Subsequently, the dried powders were macerated with ethanol 70% for 6 days. Finally, the fluid extract was concentrated by rotary evaporate apparatus under reduced pressure, and then freeze dried. 

Briefly, 500 mg standard tea extract was mixed with 70 ml sterile distilled water at 50°C. Then, 9 ml ethylic alcohol and 20 ml ethylene glycol were added to the suspension. In the next step, 100 mg benzoic sulfinide 0.05% as a sweet maker, 180 mg methylparaben 0.02%, and 20 mg propylparaben were mixed with 30 ml sterile distilled water at 80°C. After purification, 0.2 ml chloroform water was added [10].

***Determination of phenolic contents***

The amount of phenol in the hydroalcoholic extract of *C. sinensis* was determined by Folin-Ciocalteu method. Then, 0.1 ml of sample solution was mixed with 0.25 ml 1 N Folin reagent. After 5 min, 1.25 ml of 20% sodium carbonate solution was added and shaken vigorously. The absorbance of the samples was measured at 725 nm after 40 min incubation at room temperature with a double beam Perkin Elmer UV/Visible spectrophotometer. Calibration curve was obtained by standard concentrations of tannic acid. The total phenol content was expressed as equivalents of tannic acid [11].

***Determination of flavonoids contents***

The total flavonoid content was determined by aluminum chloride method. To this end, 0.5 ml of methanolic sample solution was mixed with 1.5 ml methanol, 0.1 ml of 10% anhydrous aluminum chloride in methanol, 0.1 ml of 1 M potassium acetate, and 2.8 ml distillated water. After 30 min incubation at room temperature, the absorbance of the samples was measured at 415 nm. The calibration curve was prepared by standard concentrations of methanolic solution of quercetin. The total flavonoid content was expressed as equivalents of quercetin [12].

***Mycological and polymerase chain reaction examination***

Direct examination of specimens was performed using the lactophenol cotton blue. For initial identification, the clinical samples were cultured on CHROMagar *Candida* medium (Becton Dickinson & Company, Baltimore, MD, USA) at 35°C for 48 h. All isolates were reconfirmed by polymerase chain reaction (PCR) using two primers, namely ITS1 (5′-TCC GTA GGT GAA CCT GCG G-3′) and ITS4 (5′-TCC TCC GCT TAT TGA TAT GC-3′). The PCR amplification and sequencing were performed as previously described [13]. 

A minimum of 1 ml of sample obtained from each patient was collected into a sterilized tube and mixed using a vortex mixer. Then, 0.5 ml of the sample was incubated on sabouraud dextrose agar (Difco) at 37°C for 48 h. The number of colony forming units (CFU) per milliliter of sample was counted by visual inspection. Colony counts were performed for all 22 isolates of patients both before and after the treatment.

***Treatment process***

After proving the *Candida* infection based on the clinical and mycological criteria, the patients were included in the treatment process [14]. To this end, the patients were randomly assigned into two groups, namely nystatin (n=11) and green tea (n=11), receiving nystatin suspension 100,000 u/ml (Emad Pharmaceutical Co., Esfahan, Iran) and 15 ml green tea mouthwash (0.5%) four times a day, respectively. The lesion size (length and width) was measured on the 1^st^, 7^th^, and 14^th^ days using a caliper.

***Statistical Analysis***

The Student’s 𝑡-test was used to analyze the quantitative data (e.g., age and duration of prosthesis). The comparison of the two groups in terms of gender and *Candida* colony count was accomplished using Fisher’s exact test and Mann-Whitney U test, respectively. Furthermore, the CFU/ml reduction was analyzed by repeated measures ANOVA. The present study was recorded in the clinical trial system with a code of IRCT2017060334314N1. The research protocol was approved by the Ethics Committee of Mazandaran University of Medical Sciences (IR.MAZUMS.REC.95.1560).

## Results and Discussion

Out of 22 patients, 16 (72.7%) cases were female (*P*=*0.3354*). The green tea mouthwash group included 2 males and 9 females, and the nystatin group consisted of 4 males and 7 females with the mean age of 65 ± 11.3 years (*P*=*0.951*). In terms of the education level, 54.6% and 63.6% of the patients in the green tea and nystatin groups had elementary education, respectively (*P=0.370*).

The mean durations of using dental prosthesis in the green tea and nystatin groups were 6.8 and 6.8 years, respectively (*P*=*0.718*). Furthermore, 63.6% and 81.85% of the patients in the green tea and nystatin groups cleaned the dental prostheses with water, respectively (*P*=*0.325*). The results revealed no statistically significant difference between the two groups in terms of demographic information. The results of total phenol and flavonoid content using Folin-Ciocalteu method demonstrated 379 mg Tannic acid/g dry extract and 291 mg Quercetin/g dry extract, respectively.

According to the repeated measures analysis of variance (ANOVA), the mean length and width of lesions showed in green tea group decreased on the duration of treatment (*P<0.001*). As well as, the mean length and width of lesions in nystatin group demonstrated a significant difference at the time of treatment ((length, *P=0.001*) and (width, *P=0.004*)). Overall, no statistically significant difference between the two groups in terms of the mean length (*P=0.179*) and width (*P*=0.390) of lesions. Although, the mean of *Candida* colony showed significant differences in these two groups before and after the treatment, no statistically significant result was found in the mean of *Candida* colony between the nystatin and green tea group after the treatment. (*P=0.193*). 

Based on the results, 56% and 44% of the denture stomatitis cases were caused by *C. albicans *and non-*albicans Candida. *Among the non-*albicans Candida, C. glabrata* (n=5, 20%) and *C. tropicalis* (n=5, 20%) were the leading agents, followed by *C. krusei* (n=1, 4%). Additionally, co-infection due to *C. albicans, C. glabrata*, and *C. tropicalis* was reported in three cases.

It has been reported the side effects of antifungal drugs in oral candidiasis [15]. Therefore, the need for the use of effective herbal drugs with less side effects is imperative. Some plant extracts with many biological activities for the treatment of various diseases can be considered as an alternative treatment. *Camellia sinensis* is a plant widely grown in the north of Iran. The leaves of this plant have been widely consumed by the Iranian people. There are some reports on the antifungal activity of this plant against *Candida* species [16, 17]. 

The current study was the antifungal activity of green tea against the tested oral candidiasis and compared with nystatin which have been prescribed as the first line of oral candidiasis treatment. Green tea as a plant extract may be a good substitute for the treatment of denture stomatitis due to its availability, minimum side effects, and low cost. 

Regarding the prevalence of species, six *Candida* species were identified in the isolated samples. *Candida albicans *was the most frequently isolated in both groups followed by *C. glabrata*, *C. tropicalis,* and *C. krusei*. These results are consistent with the data published by Marcos-Arias et al. [18] and Zomorodian et al. [19]. *Candida* species can colonize on denture and result in the formation of biofilms, which have a major role in the development of denture stomatitis. 

Green tea not only has antimicrobial activity but also it has anti-inflammation, anti-oxidant, anti-mutation, and anti-diabetic properties, which can play an important role in the treatment of erythema and mucosal inflammation [18, 20]. These findings corroborate the research by Najafi et al., [16] which evaluated the healing action of green tea and nystatin on denture stomatitis and concluded that green tea can be as effective as nystatin. Some authors also demonstrated antifungal activity of green and black tea and that the antifungal effect was directly proportional to the concentration [5, 21]. It is recommended to perform biochemical studies to promote the use of green tea as an antifungal agent. 

## Conclusion

This study demonstrated that green tea mouthwash can decrease the burden of *Candida* species in patients with denture stomatitis. This result might be due to the fact that green tea is effective against a wide range of oral microorganisms owing to its anti-oxidant and anti-inflammatory nature. There are some limitations in this study consisting the number of patients and the study period, these results indicate that further studies are needed to investigate the effects of green tea to decrease denture-related stomatitis.
